# Endocannabinoid Modulation of Stimulus-Specific Adaptation in Inferior Colliculus Neurons of the Rat

**DOI:** 10.1038/s41598-017-07460-w

**Published:** 2017-08-01

**Authors:** C. Valdés-Baizabal, G. G. Parras, Y. A. Ayala, M. S. Malmierca

**Affiliations:** 1 Auditory Neuroscience Laboratory, Institute of Neuroscience of Castilla y León, Calle Pintor Fernando Gallego 1, 37007 Salamanca, Spain; 2 The Salamanca Institute for Biomedical Research (IBSAL), 37007 Salamanca, Spain; 3 Department of Biology and Pathology, Faculty of Medicine, Campus Miguel de Unamuno, University of Salamanca, 37007 Salamanca, Spain; 4 Present Address: Instituto de Neurobiología, Universidad Nacional Autónoma de México, Querétaro, Mexico

## Abstract

Cannabinoid receptors (CBRs) are widely distributed in the brain, including the inferior colliculus (IC). Here, we aim to study whether endocannabinoids influence a specific type of neuronal adaptation, namely, stimulus-specific adaptation (SSA) found in some IC neurons. SSA is important because it has been found as early as the level of the midbrain and therefore it may be a neuronal correlate of early indices of deviance detection. Furthermore, recent studies have demonstrated a direct link between SSA and MMN, that is widely used as an outcome measure in a variety of human neurodegenerative disorders. SSA is considered a form of short-term plasticity, and CBRs have been shown to play a role in short-term neural plasticity. Therefore, it is reasonable to hypothesize that endocannabinoids may play a role in the generation or modulation of SSA. We recorded single units in the IC under an oddball paradigm stimulation. The results demonstrate that cannabinoid agonists lead to a reduction in the neuronal adaptation. This change is due to a differential increase of the neuronal firing rate to the standard tone alone. Furthermore, we show that the effect is mediated by the cannabinoid receptor 1 (CBR1). Thus, cannabinoid agonists down-modulate SSA in IC neurons.

## Introduction

Anatomical and physiological studies have demonstrated that endocannabinoids (ECBs) modulate neural processing in sensory systems^1–4^, including the auditory system. Indeed, many nuclei in the auditory brainstem and midbrain, such as cochlear nuclei (CN), superior olivary complex (SOC) and inferior colliculus (IC), express cannabinoid receptors (CBRs). Moreover, previous *in vitro* studies have shown that ECBs modulate electrophysiological properties in CN^5, 6^ and SOC neurons^7^. For example, in CN cartwheel neurons, ECBs selectively suppress glutamatergic synapses^6^, while endocannabinoid signaling attenuates both glycinergic and glutamatergic postsynaptic currents in SOC neurons^7^. IC neurons express CBRs^8, 9^ and therefore, ECBs probably modulate their responses, but, to date, effects of ECBs in the mammals IC have not been electrophysiologically studied.

The canonical view of the mechanism of action of the CB system (ECBs, and receptor types 1 and 2, CBR1 and CBR2) is that the ligand is released by a postsynaptic neuron and acts as a retrograde messenger on receptors located on presynaptic terminals. Both receptor types are coupled to G-proteins^5^. CB1Rs are expressed predominantly in the mammalian central nervous system while CB2Rs are located mainly in the peripheral nervous system and immune tissues. ECBs are produced on demand in an activity-dependent manner. When a neuron is stimulated synaptically, ECB synthesis is initiated via a Ca^2+^-dependent activation of ECB-synthesizing enzymes, and the ECBs are then released into the synaptic cleft. The released ECBs act as retrograde messengers at central synapses^10^, resulting in the activation of presynaptic CB1Rs^4, 11^, which attenuates Ca^2+^ influx into the presynaptic terminal, blocking vesicle fusion, and thus decreasing transmitter release^12^. This retrograde mechanism is called depolarization-induced suppression of inhibition^13, 14^ or depolarization-induced suppression of excitation^15^, depending on whether the ECBs act on an inhibitory or excitatory input^16^.

Here, we aim to examine whether ECBs influence a specific type of neuronal adaptation found in the IC and beyond along the auditory pathway, namely, stimulus-specific adaptation (SSA). SSA in IC^17–25^ and primary auditory cortex (A1)^26–33^ occurs mainly in early responses (20–40 ms), so it may be a neuronal correlate of early indices of deviance detection^30, 31, 34–36^. However, SSA in non-primary AC occurs within, and beyond, the MMN time window^33^. Hence a direct link between SSA and MMN has already been established.

SSA is elicited under oddball paradigm stimulation and consists of a rapid and pronounced decrement of neural responsiveness to trains of identical stimuli (standard stimuli), even at low repetition rates on the order of seconds^24, 26^. SSA neurons recover their responsiveness whenever certain stimulus parameters are changed (deviant stimuli)^17^. SSA also occurs in other sensory systems^37–39^. Over the last decade, a series of studies from our lab has reported the principal electrophysiological properties and the details of organization of SSA in auditory midbrain neurons^17–25, 30–32^. SSA in the IC is mainly a property of non-lemniscal IC neurons (the IC cortical regions) and SSA is not homogeneously distributed throughout the neuron’s frequency response area, such that higher levels of SSA are found at low intensity levels and at the high frequency edges of the neurons´ receptive fields. SSA is modulated by acetylcholine^25^ and GABA-A mediated inhibition^39^. Moreover, SSA in the IC is not a property inherited from the AC^32, 40^ as originally suggested^26^.

Because SSA is considered a form of short-term plasticity^41^ and ECBs have been shown to play a role in short-term plasticity^4, 7^, it is plausible that ECBs play a role in the generation or modulation of SSA. Moreover, since cannabinoid receptors are expressed in the IC, we hypothesize that cannabinoid drugs modulate SSA responses of the IC neurons. To document a functional role of endocannabinoid system on IC neuronal activity and, more specifically, to discover how ECBs affect SSA, we performed two sets of complementary experiments; namely, intravenous and intracerebral microiontophoretic applications of cannabinoid drugs while testing for SSA. The results demonstrate that the endocannabinoid system down-modulates SSA responses in rat IC neurons.

## Results

**Figure 1:**
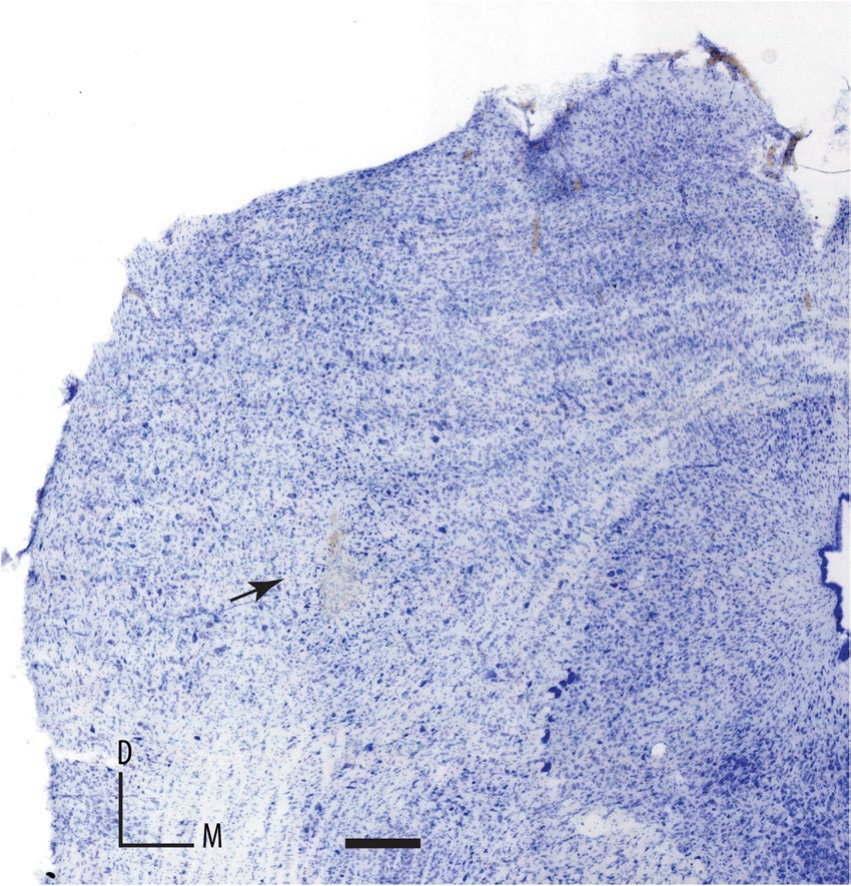
Photomicrograph of a coronal section through the rostral cortex of the IC showing a typical electrolytic lesion of a recording site for a neuron with a high CSI value. Scale bar, 1 mm. M, medial; D, dorsal.

To determine the influence of ECBs on SSA, we recorded the response of 154 well-isolated IC neurons under the oddball paradigm before, during and after the application of the CB1R agonists anandamide (n = 50, *i.v*.), and O-2545 (n = 40, microiontophoretically), and the CB1R antagonist AM251 (n = 49, *i.v*.) as well as a drug cocktail made of the CB1R agonist anandamide and CB1R antagonist AM251 (n = 15, *i.v*.). (Anandamide and AM251 were administered *i.v*. because they are not water soluble and so could not be administered iontohoretically.) Because previous studies have shown that SSA is maximum in the cortical regions of the IC^17–19^, we specifically attempted to record neurons from these regions. The subsequent histological verification of the recording sites, marked by lesions showed that 67% were in the rostral cortex (e.g., Fig. 1) and 33% were in the lateral cortex of the IC. Overall, there were no differences between the effects obtained in these two cortices, and so the data were pooled into a single sample. We also analyzed the effect of the drugs on some properties other than SSA, such as spontaneous activity and spectral sensitivity (FRA).

### The effect of anandamide on firing rate and SSA level

**Figure 2:**
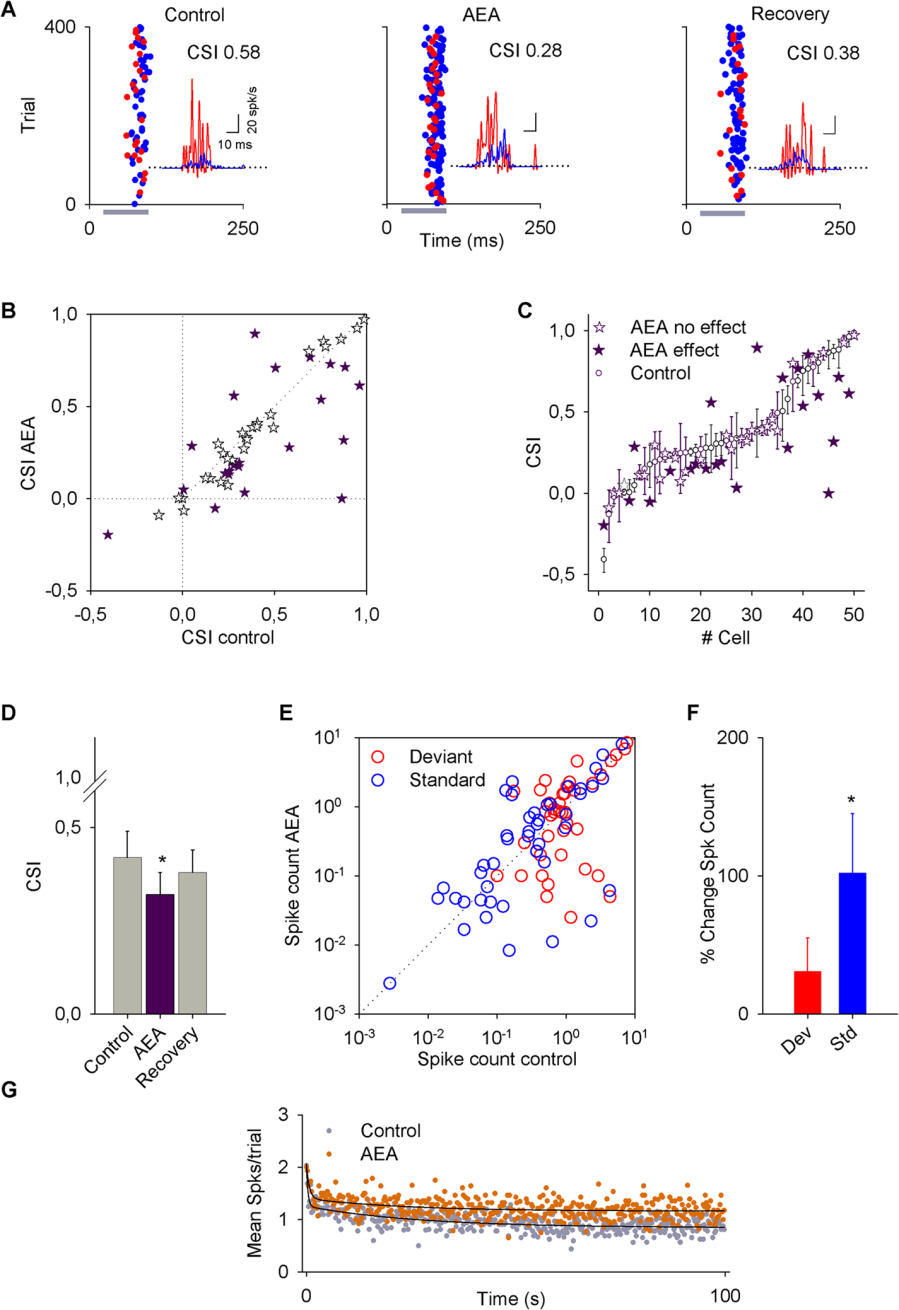
Effect of anandamide (AEA) on the activity of inferior colliculus (IC) neurons. (**A**) Typical recording of an IC neuron under an oddball stimulation paradigm before (control), during (AEA) and after (recovery) an i.v. application of AEA. (For this neuron, the standard frequency was 2105 Hz and the deviant frequency was 1724 Hz.) Application of AEA decreased the CSI from 0.58 to 0.28. For this and subsequent figures, the gray horizontal bars indicate the duration of sound stimulation and asterisks indicate a P-value less than 0.05. The insets are PSTHs that represent the mean response to the oddball sequence, in all conditions showing a significantly larger neuronal response to the deviant tone (red) than to the standard (blue). (**B**) Scatter plot of the CSI in control condition versus drug application. It can be seen that the CSI of most neurons decreases. (**C**) Bootstrapping analysis for each neuron. White dots indicate the control CSI and stars indicate the AEA application (purple stars: significant change; white stars: no change). (**D**) Bars represent the average value ± SE of the change for the population that had significant changes in the CSI. (**E**) Scatter plot of the spike count in the control condition versus AEA application for standard (blue dots) and deviant (red dots) stimuli. (**F**) Percent change in the responses to deviant and standard stimuli (vertical bars represent the % change ± SE). The AEA significantly increases the response to the standard stimulus. (**G**) Time course of adaptation for the mean response to the standard frequency for each position (time) in the oddball sequence of neurons significantly affected by anandamide. The baseline (gray circles) and anandamide data (orange circles) had fast and slow decay components and a steady-state component that were fitted by a double exponential function (black lines).

Figure 2A shows a typical example of a single-unit response before, during and after drug application. The injection of anandamide elicited a significant increase in the response to standard stimuli that resulted in a significant decrease of the CSI, from 0.58 to 0.28. For most neurons, as it is the case shown in Fig. 2A, the firing rate was only partially recovered. However, we considered an almost or partial recovery when the firing rate values were not significantly different to those in the control condition.

An analysis of the CSI of the whole sample (n = 50) showed that there is a marked tendency toward a decrease of the CSI after drug application (Fig. 2B). To determine the significance of the effects, we performed a bootstrapping analysis evaluating the effect of anandamide on each individual neuron (Fig. 2C). This analysis showed that anandamide affected 23/50 neurons, and the population *t-test* showed that overall anandamide decreased the CSI from 0.42 ± 0.07 to 0.32 ± 0.06 (P = 0.048; Fig. 2D). In this group of neurons, the CSI decreased in 16/23 and increased in 7/23 (details provided in Table 1).

**Table 1:** Summary of effects of cannabinoid drugs on the CSI.

Drug	n	CSI		
		# neurons with significant change	Total change	Segregated changes
Anandamide (agonist)	50	23	↓ 0.42 to 0.32 (P = 0.048) (parametric test)	16/23 ↓ 0.51 to 0.27 (P < 0.001) (non-parametric test)
				7/23 ↑ 0.22 to 0.44 (P = 0.008) (parametric test)
O-2545 (agonist)	40	13	↓ 0.53 to 0.29 (P = 0.012) (parametric test)	11/13 ↓ 0.61 to 0.31 (P = 0.003) (parametric test)
				2/13 ↑ 0.21 to 0.4 (no statistical analysis)
AM251 (antagonist	49	7	↑ 0.17 to 0.43 (P = 0.017) (parametric test)	N/A*

*N/A Does not apply.

Next, we analyzed how the firing rate of the neurons showing a change in their CSI (n = 23) was affected. The firing rates for both standard and deviant stimuli are plotted in Fig. 2E. The spike count increased significantly in response to standard stimuli (by 102 ± 43%, P = 0.013), while the spike count in response to deviant stimuli was unchanged. The bar plots in Fig. 2F show the percent change in the responses to deviant and standard stimuli after anandamide application.

In order to study the dynamics of adaptation to the repetitive stimuli, we averaged responses to standard stimuli across recordings for every trial within the sequence. The response to the standard frequency was fit by a double exponential function under the baseline (R^2^ = 0.65) and anandamide (R^2^ = 0.43) conditions, displaying a rapid and a slow decay as well as a steady state component (Fig. 2G). Anandamide increased the steady-state component of the response from 0.84 spikes per trial to 1.16 (95% CIs) without affecting either the timing or the magnitude of the fast (baseline, τ_r_ = 0.37 trial, A_r_ = 0.77 spikes per trial; anandamide τ_r_ = 0.67 trial, A_r_ = 0.65) or slow (baseline, τ_s_ = 27.23 trial, A_s_ = 0.41 spikes per trial; anandamide τ_s_ = 23.68 trial, A_s_ = 0.24) components of the adaptation (Fig. 2G).

### The effect of O-2545 on firing rate and CSI

**Figure 3:**
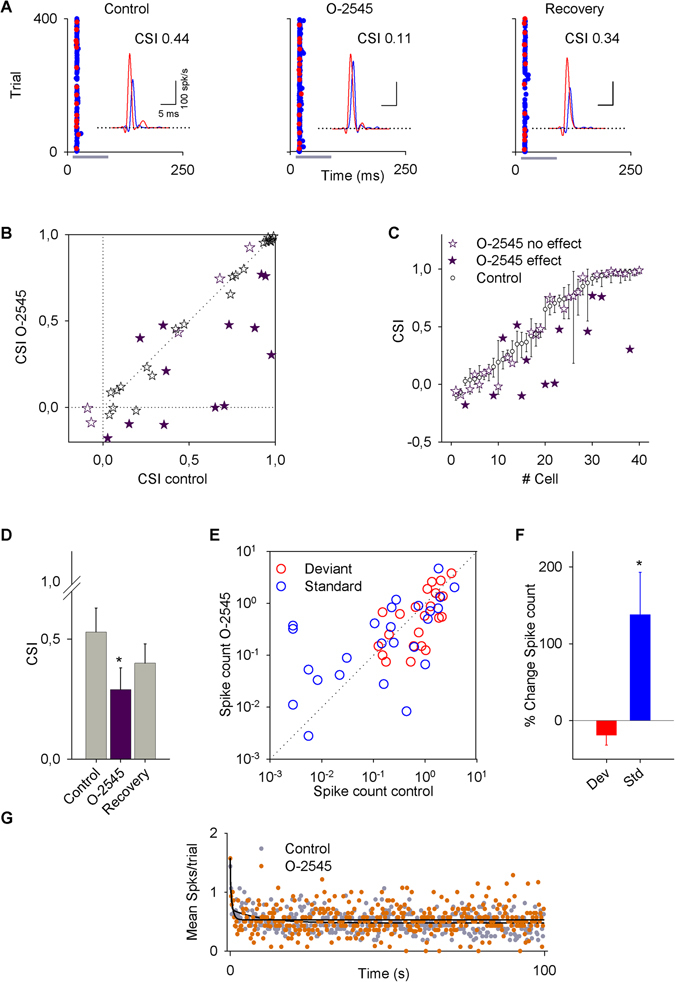
Effect of O-2545 on the activity of IC neurons. (**A**) Typical recording of an IC neuron under an oddball stimulation paradigm before (control), during (O-2545), and after (recovery) microiontophoretic application of O-2545. (For this neuron, the standard frequency was 34544 Hz and the deviant frequency was 28284 Hz.) Insets represent the PSTH of the response in which the CSI decreased from 0.44 to 0.11 and recovered to 0.34. (**B**) Scatter plot of the CSI in the control condition versus O-2545 application. The CSI of most of the neurons decreased. (**C**) Bootstrapping analysis for each neuron recorded in control condition (white dots) and with O-2545 application (purple stars: significant change; white stars: no change). (**D**) Bars represent the averaged CSI ± SE of the neurons in which there were significant changes. (**E**) Scatter plot of the spike count in control condition versus drug application for deviant (red dots) and standard (blue dots) stimuli. (**F**) Bars represent the percent change in the spike count for deviant (red bar) and standard (blue bar) stimuli. Vertical bars represent the % change ± SE. (**G**) Time course of adaptation of O-2545. The baseline (gray circles) and O-2545 data (orange circles) had fast and slow decay components and a steady-state component that were fitted by a double exponential function (black lines).

Since anandamide was administered intravenously instead of microiontophoretically due to its poor water solubility, the changes observed might reflect not only direct effects on IC neural activity but also actions on other brain regions projecting directly or indirectly to the IC neurons. To address this issue we conducted a second experimental series using the microiontophoretic injection of 25 mM O-2545 while recording single unit responses from a total of 40 IC neurons. A typical example of a single-unit recording is illustrated in Fig. 3A under the control condition, after drug application and during recovery. In this particular neuron, the O-2545 drug significantly decreased the CSI from 0.44 to 0.11 followed by a recovery to basal levels after the injection was terminated.

We first analyzed the average change in CSI for the entire population (n = 40). This analysis revealed that overall the CSI showed a marked decrease (Fig. 3B). As with anandamide, we performed a bootstrapping analysis to determine the effect on each individual neuron (Fig. 3C). This analysis showed that the CSI significantly decreases from 0.53 ± 0.1 to 0.29 ± 0.1 (n = 13; P = 0.012) and thus, the reduction is about 50% (Fig. 3D). Table 1 contains the details of the effect of O-2545.

Figure 3E shows the firing rate of the neurons with a significant change in CSI (n = 13). O-2545 significantly increased the response to the standard stimulus by 138 ± 55% (P = 0.037, Fig. 3F) without changes in the firing rate to the deviant stimulus.

We also analyzed the effect of O-2545 on the time course of the response to the standard stimulus for the neurons with a significant change in their CSI. The dynamics of the response to the standard frequency was fit by a double exponential function under the baseline (R^2^ = 0.38) and O2545 (R^2^ = 0.26) conditions, displaying a rapid and a slow decay as well as a steady state component (Fig. 3G). O-2545 increased the response during the steady-state component of the response from 0.48 spikes per trial to 0.53 (95% CIs) without affecting either the timing or the magnitude of the fast (baseline, τ_r_ = 0.21 trial, A_r_ = 0.74 spikes per trial; O-2545 τ_r_ = 0.003 trial, A_r_ = 0.34) or slow (baseline, τ_s_ = 9.58 trial, A_s_ = 0.24 spikes per trial; O-2545 τ_s_ = 0.63 trial, A_s_ = 0.70) components of the adaptation (Fig. 3G). O-2545 seems to change the variance more than anandamide.

### AM251 decreases the firing rate but not the CSI

**Figure 4:**
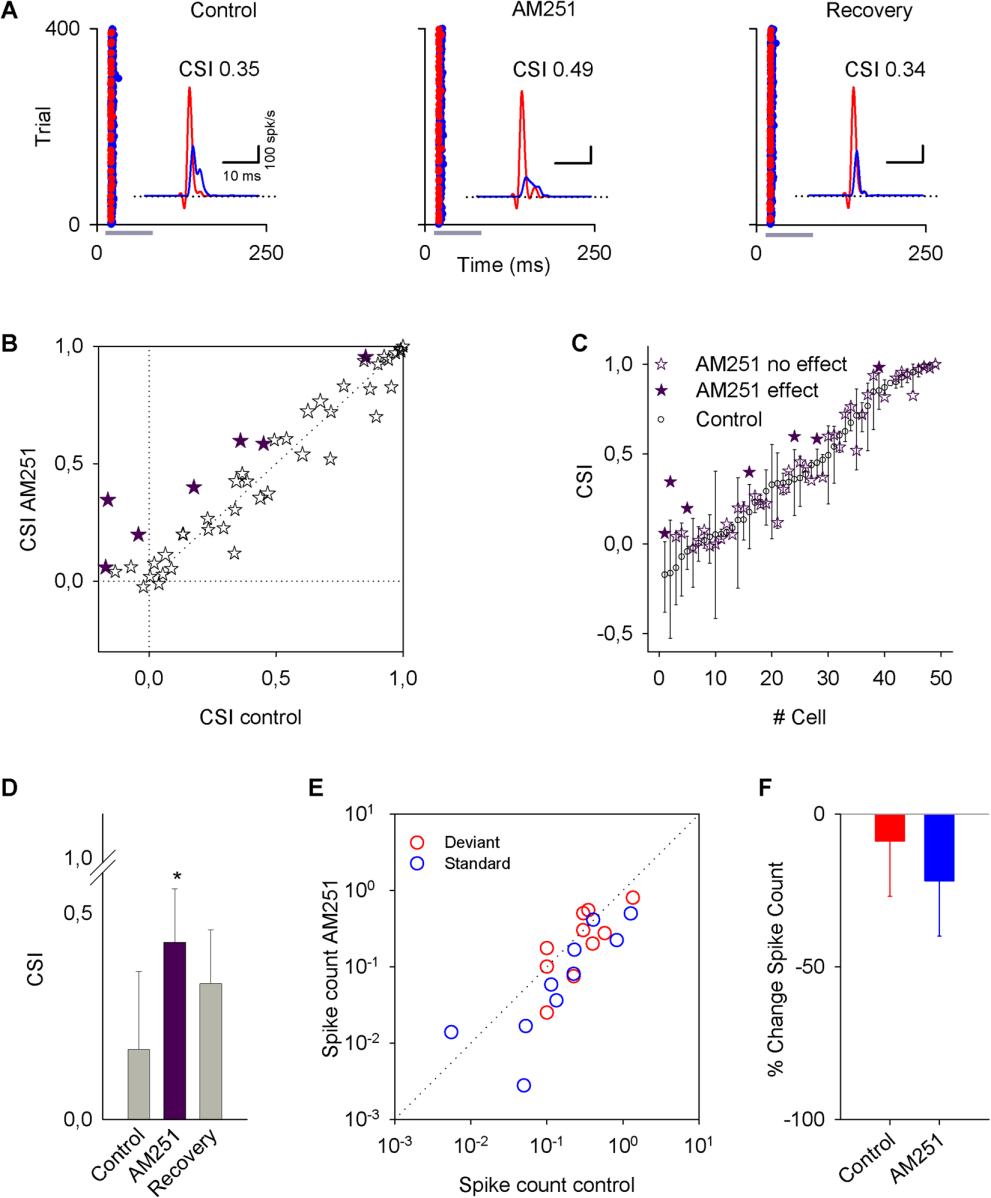
Effects of AM251 on the activity of IC neurons. (**A**) Typical recordings of an IC neuron under an oddball stimulation paradigm before (control), during (AM251), and after (recovery) an i.v. application of AM251. (For this neuron, the standard frequency was 8574 Hz, and the deviant frequency was 10472 Hz.) The insets show the PSTH of the responses. (**B**) Scatter plot of the CSI in control condition versus AM251 application. (**C**) Bootstrapping analysis showing that AM251 produced significant changes in the CSI only in 7/49 neurons. (**D**) Bars represent the average of the CSI values ± SE in control condition, AM251 application and recovery. (**E**) Scatter plot of the firing rate showing the spike count for deviant and standard frequencies in control condition versus AM251 application. (**F**) Bars show the percent change in the spike count averaged for deviant and standard stimuli by AM251 application. There is a tendency for a decrease in the firing rate for both frequencies. Vertical bars represent the % change ± SE.

AM251 was applied to a total of 49 IC neurons. An example of its effect on the single-unit response is shown in Fig. 4A. The application of AM251 on IC neurons did not show a clear tendency to change the CSI at the population level (Fig. 4B). However, when a bootstrapping analysis was performed (Fig. 4C), 7/49 neurons displayed a significant change, with an increase in the CSI from 0.17 ± 0.2 to 0.43 ± 0.13 (Fig. 4D; Table 1). Non-significant changes were observed in the firing rate to deviant and standard stimuli (Fig. 4E and F). Although the effects on the firing rate of the neurons are not significant, there is a tendency for a decrease in the firing rate to the standard stimulus (36%; P = 0.105), leading to changes in the CSI.

**Figure 5:**
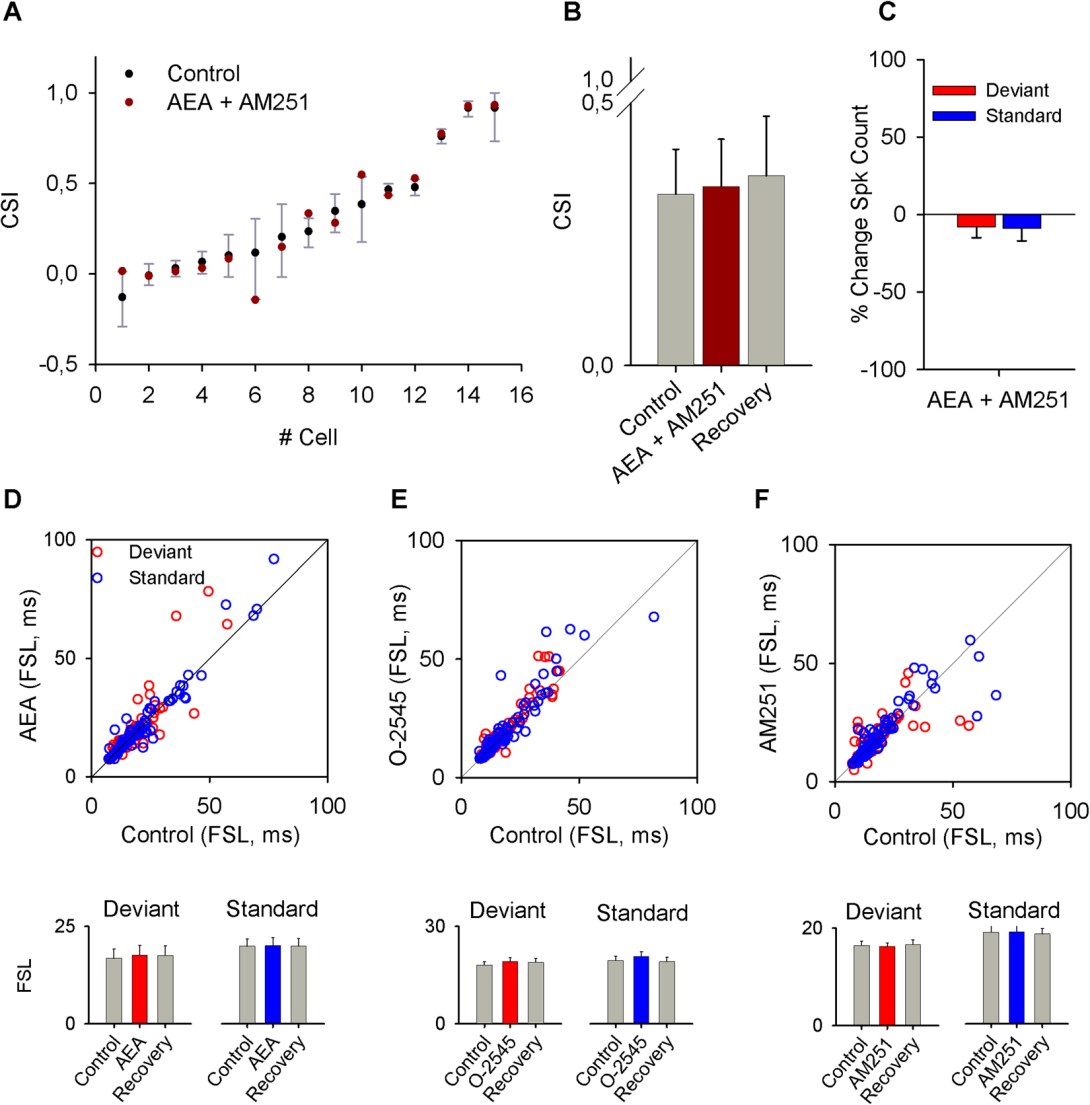
Effects of the co-application of AEA and AM251. (**A**) Bootstrap analysis of the effect of the combination of agonist plus antagonist showing that co-application did not produce effects on the CSI of the IC neurons. (**B**) Bar graph representation of the effect of the combination of drugs on the average value of CSI. (**C**) Percent change of the spike count in response to standard and deviant frequencies after application of agonist plus antagonist. (**D**–**F**) Scatter plots that represent the FSL in control condition versus drug application, for AEA, O-2545 and AM251 respectively. Below are bar plots of the average values of FSL for deviant and standard stimuli in control condition, with drug application and during recovery.

Next, we injected a drug cocktail made of anandamide and AM251. Its application produced no significant changes in any of the measured parameters (n = 15), including CSI (Fig. 5A and B) and spike counts (Fig. 5C). This result demonstrates that the effects we observed were specifically mediated by CB1R.

### Effect of cannabinoid drugs on latency, spontaneous activity and other properties of IC neurons

Neither the agonists nor the antagonist affected the first spike latency (FSL), which was shorter in response to the deviant stimulus compared to the standard stimulus, as previously described^17, 39^ (Fig. 5D–F). A small percentage of all recorded neurons (23%, 32/139) exhibited spontaneous activity (0.51 ± 0.16 spikes per second). There were no detectable effects on the spontaneous activity after application of the agonists. However, when the antagonist AM251 was applied, the spontaneous activity significantly increased by 7 ± 4% (P = 0.023). Finally, the properties of several different parameters including FRA shape, BF, CF, threshold, Q_10_, Q_30_, BW10 and BW_30_ analyzed in 118 neurons, remained unchanged.

## Discussion

The results of this study demonstrate that ECBs exert an effect on SSA in non-lemniscal IC neurons of the rat. When IC neurons are stimulated under the oddball paradigm, cannabinoid agonists affect the neuronal firing of IC neurons mostly increasing the responses to standard stimuli but not the response to the deviant one. This differential effect leads to a decrease in the level of SSA. Based on the pharmacological properties of the agonists and antagonists, we conclude that the effect of anandamide is specifically mediated by CB1 receptors, because the SSA levels did not change with the co-application of the agonist plus antagonist.

The retrograde modulation of inhibitory and excitatory inputs by cannabinoids has been previously described for both glutamatergic and GABAergic synapses in the auditory pathway^5^. It is well known that the IC receives excitatory and inhibitory inputs and that the interplay between them determines the activity of IC neurons^42^. These inputs may be under the influence of a delicate neuromodulation. It is also known that IC neurons express CBRs, but their functional expression and their effects on SSA remained unknown.

Here we report for the first time that the cannabinoid agonists anandamide (an endogenous CB1R agonist) and O-2545 (a high affinity synthetic CB1R agonist) decrease neuronal adaptation in the IC as measured by the CSI, i.e., the SSA index^17, 26^. In the IC, this effect is due to a differential increase in the spike count to repetitive stimuli. There are at least three possibilities that could account for these results. The first is based on the well-known mechanism through which cannabinoids act on presynaptic receptors. The second depends on the combined action of cannabinoids and other neuromodulatory substances such as acetylcholine. A potential third mechanism requires the presence of postsynaptic CBRs in addition to the known presynaptic receptors. Although it is currently unknown whether mammalian IC neurons express postsynaptic CBRs, we cannot rule out this possibility. These three mechanisms are not mutually exclusive. In the following we discuss each possibility in the context of our main results.

**Figure 6:**
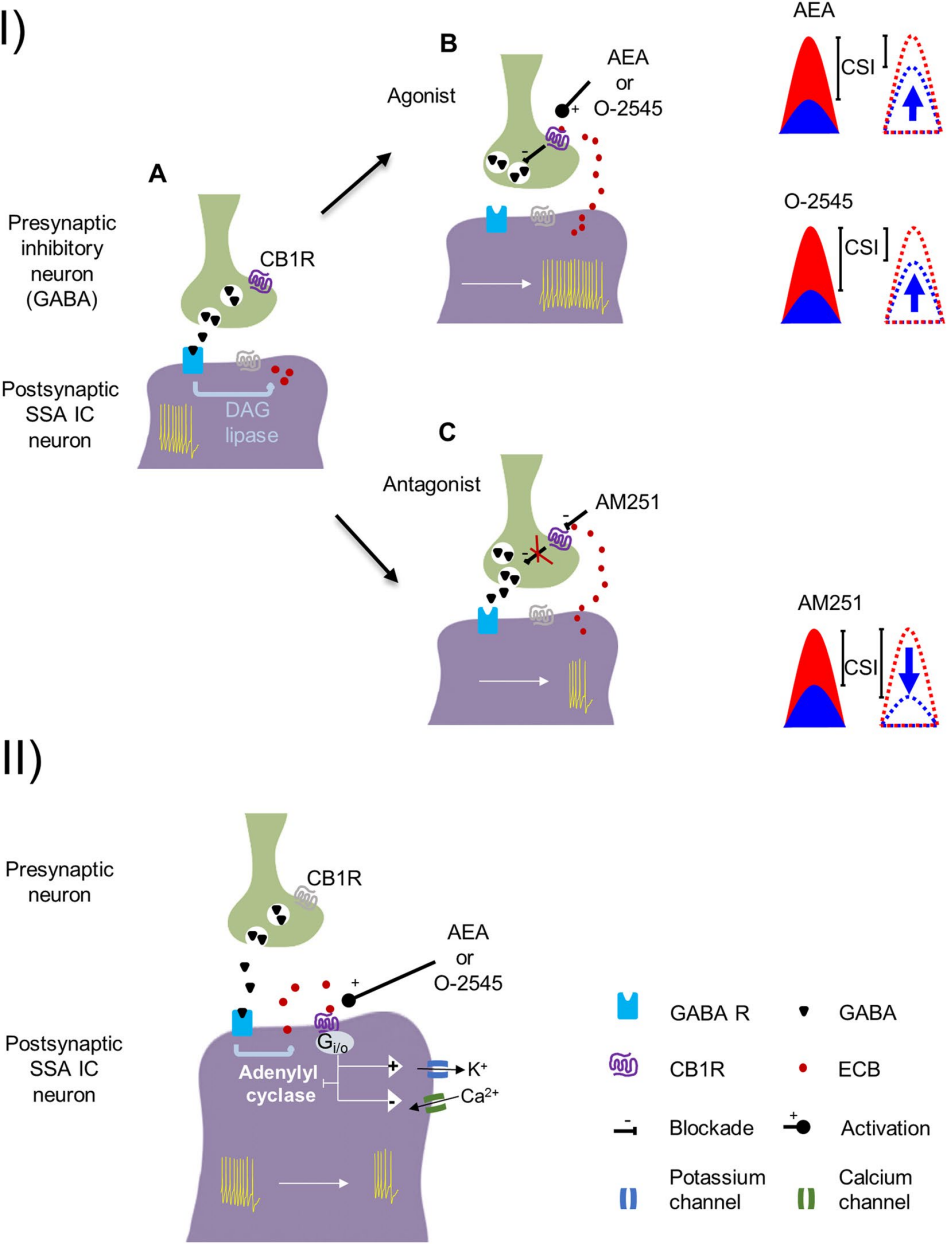
Schematic representation of hypothetical action mechanisms of cannabinoids on IC neurons that exhibit SSA. (**I**.A) An inhibitory input (probably GABAergic, green) contacting a postsynaptic IC neuron (purple). Basal activity is shown as action potentials (yellow) in the postsynaptic neuron. GABA release affects the activity of the neuron by acting on postsynaptic receptors. (**I**.B) Agonist drugs that activate presynaptic CB1R on the inhibitory terminal lead to an increase in the firing rate of the postsynaptic neuron due to a decrease in the GABA release. Both agonists produce an increase of the firing rate in response to the standard frequency; thus, the CSI of the postsynaptic SSA IC neuron decreases. We cannot rule out the possible involvement of other neuromodulatory substances in the final result. (**I**.C) Injection of the CB1R antagonist AM251 producs a blockade of the basal ECB and/or the constitutive activity of the receptors, hence GABA is released, decreasing the firing rate for standard stimuli that results in a CSI increase. (**II**) If CBRs are located in the postsynaptic neurons, their activation should promote both an inhibition of adenylyl cyclase, and a change in the open probability of ionic channels (K^+^ and Ca^2+^) that would lead to a decrease in neuronal activity.

Based on well-established mechanisms of cannabinoid drugs in other parts of the brain, anandamide and O-2545 could be acting on CB1Rs expressed on presynaptic inhibitory neurons, leading to the observed increases in neuronal responses to repetitive stimuli. Previous studies have shown that GABA_A_-mediated inhibition plays a role in shaping SSA by acting as a gain control system^23, 39^. Our results suggest that the postsynaptic neurons from which we recorded are likely to receive inhibitory inputs (probably GABAergic) expressing CB1Rs^43^. These inputs would normally inhibit the postsynaptic neuron, but the application of the cannabinoid agonists would activate the CB1Rs on the presynaptic terminals, decreasing GABA release and so increasing the activity of the postsynaptic neuron (Fig. 6.I). We cannot rule out an effect mediated by glycinergic inputs, but this is unlikely because results from our lab have shown that glycine-mediated inhibition has only a weak effect, if any, on SSA^44, 45^. Further, glycineric receptors are expressed mainly in the ventral part of the central IC^43, 46^ where SSA is almost negligible^17, 19, 24^.

A wealth of data supports the idea that modulation of GABA-mediated inhibition is a common mechanism in the central nervous system. It is known that endocannabinoids modulate GABA release in many CNS regions, including the hippocampus, basal ganglia, cerebellum and brainstem^12, 14, 47–51^. Likewise, anatomical studies have demonstrated that high levels of CB1R mRNA and immunoreactivity are associated with GABAergic neurons^12, 47, 52^. Hence, a GABA suppression-mediated mechanism is a plausible explanation for the action of cannabinoid agonist drugs on SSA in IC neurons (Fig. 6.I). The specific increase on standard response might be explained by an activity-dependent CBRs-activation. Considering that SSA can be accounted for by differential activation of afferent inputs (Duque *et al*.^21^), repetitively activated afferents by the standard frequency would lead to a ‘larger’ release of ECBs at their synapse with the IC neurons than those released by inputs activated by deviant frequencies. The CBRs activation would in turn decrease the inhibition locally recruited by standard-activated frequency channels acting on SSA neuron. In this context it is worth to mention that a growing body of evidence suggests that perturbations in GABAergic synaptic transmission, such as reduced CB1R expression, are linked to schizophrenia^51, 53^. Since MMN is altered in schizophrenia^54^ and SSA may be a neuronal correlate of MMN^27–29^ our study may open new avenues for future studies on the relationship between ECBs, SSA, MMN and schizophrenia. We did observe decreases in firing to the repetitive stimulus in a few neurons; this result could be explained by an effect on excitatory (glutamatergic) inputs expressing CB1Rs. Agonists would promote a blockade of excitation through action on CB1R receptors expressed on glutamatergic presynaptic neurons, for this small population of neurons. There is electrophysiological evidence that CBR activation inhibits glutamate release in many brain regions, including the Purkinje neuron-parallel fiber synapse in the cerebellum and also at synapses in the striatum, midbrain periaqueductal gray, and nucleus accumbens^15, 49, 55, 56^. This possibility could explain the mixed effects that we have observed when we applied anandamide or O-2545. Also it is plausible that ECBs modulate both excitatory and inhibitory inputs and that the final increase or decrease of the firing rate depends on the ratio of ECB modulation to both inputs.

A second possibility that could explain some of our results is synergistic activity of the ECB system and other neuromodulators. We collected a large sample of neurons (154), but only a relatively small number showed significant changes in the CSI. It is possible that CB1Rs are differentially expressed in presynaptic neurons and that there is a population that does not express CB1Rs. But it is also possible that the endocannabinoid system, although functionally expressed, requires other modulatory substances to exert its effects. Interestingly, and similar to what we observed here with CB1R, previous work from our lab has demonstrated a differential increase in the response to a standard stimulus is also elicited by the activation of cholinergic (muscarinic) receptors^25^ such that SSA is reduced by ACh blockade. Moreover, a recent psychopharmacological study^57^ to test specific and formal predictions about the effect of cholinergic manipulations on MMN and repetition suppression has shown that by assigning ACh the role of signaling sensory precision, its augmentation can reduce adaptation to surprising stimuli in sensory cortices, i.e., MMN. We cannot confirm or rule out that the previously reported cholinergic effect on SSA^25^ and MMN^57^ is mediated by the CB1R activation, but many *in vitro* studies have demonstrated a close functional relationship between cannabinoids and acetylcholine. Co-activation of glutamate and acetylcholine receptors increases the release of ECBs^58, 59^. Further, co-treatment with glutamate and carbacol (a cholinergic agonist) stimulates the anandamide biosynthesis pathway in primary cultured cortical neurons^58^. Patch clamp recordings in hippocampal slices have demonstrated that the activation of cholinergic neurons (through muscarinic receptors) and retrograde signaling by ECBs act cooperatively to regulate GABAergic transmission through the blockade of CB1Rs or M2-type mAChRs, decreasing the probability of GABA release^50^. At the cellular level, this mechanism is mediated by cAMP/PKA (cyclic adenosine monophosphate/protein kinase A). It is known that CB1Rs are coupled to a G_i/o_ protein that induces the inhibition of cAMP with consequent non-activation of PKA. In hippocampal synapses, the decrease of PKA contributes to the decrease of ECB-dependent GABA release^50^. It is also possible that the ECB system requires the presence of other neuromodulator substances such as dopamine or serotonin to produce its effects. Future studies are necessary to test this hypothesis.

Finally, a third possible CB1Rs activation mechanism would involve the postsynaptic expression of cannabinoid receptors in IC neurons. In this case, since CB1Rs are coupled to G_i/o_ protein, one would expect that their activation would promote the inhibition of adenylyl cyclase, an increase in the open probability of potassium channels and a decrease in the open probability of calcium channels. Under this scenario, the expected effect on neuronal firing rate would be a decrease (Fig. 6.II). Although, this effect was not the main observation in our data set, there was a small subpopulation of IC neurons in which CB1R agonists led to a decrease in the spike count to standard stimuli, thus increasing the CSI (Table 1).

An important limitation of our study is that in most cases, only, partial recovery was obtained after drug application. Although we record the neurons for as long as possible, some also were lost without achieving full recovery. This could be because CB1 receptors undergo agonist-induced desensitization involving G-protein uncoupling due to phosphorylation by G-protein-coupled receptor kinases (GRKs) and receptor internalization. GRK-mediated receptor phosphorylation leads to the binding of beta-arrestins, which uncouple receptors from heterotrimeric G-proteins and target CB1 receptors for internalization in clathrin-coated vesicles^60^.

The effects elicited by the antagonist AM251 on the CSI of some neurons suggest the possibility that there is some sound-evoked endogenous ECB release, unless the CBRs are constitutively active. Such constitutive activity is a common feature in receptors with seven transmembrane segments like histamine receptors^61^, dopamine receptors^62^ and others. On the other hand, the fact that only some neurons were affected by the antagonist could indicate that the endogenous ECB release may not occur in all neurons in our anesthetized preparation^2^. A similar conclusion was reached by Dasilva and colleagues^2^ during recordings in the visual thalamus.

In summary, it is likely that ECBs have mixed and complex modulatory effects in the IC, with a major effect being a decrease in SSA. We conclude that the cannabinoid system has a role in a down-regulation of SSA in some IC neurons. The degree of modulation would depend on the strength and nature of the inputs that each neuron receives.

## Methods

### Surgical procedures

Experiments were performed on 35 adult rats (body weights: 150–250 g). All experimental procedures were carried out at the University of Salamanca using methods conforming to the standards of, and approved by, the University of Salamanca Animal Care Committee.

Surgical anesthesia was induced with a mixture of ketamine/xylazine (100 and 20 mg/kg, respectively, i.m.) and maintained with urethane (1.5 g/kg, i.p.). Supplementary urethane doses (0.5 g/kg, i.p.) were given as needed. Urethane was selected as an anesthetic because effects on multiple aspects of neural activity, including inhibition and spontaneous firing, are less apparent than with barbiturates and other anesthetic drugs^21, 25, 44^. The trachea was cannulated, and atropine sulphate (0.05 mg/kg, s.c.) was administered to reduce bronchial secretions. Body temperature was maintained at 38 °C ± 1 °C. Details of surgical preparation were as described elsewhere^17, 18, 23, 24, 39, 40, 42, 63–68^.

The animal was placed in a stereotaxic frame in which the ear bars were replaced by hollow specula that accommodated a sound delivery system. A craniotomy was performed to expose the cerebral cortex overlying the IC. A tungsten electrode^44^ (1–2 MΩ) was lowered through the cortex and used to record extracellular single unit responses in the IC. Recording sites in the IC were based on stereotaxic coordinates and physiological criteria including tonotopicity and response reliability^17, 18, 63, 65^.

### Acoustic delivery and electrophysiological recording

Stimuli were delivered through a sealed acoustic system^17, 19–21, 25^. Pure tone bursts were delivered to the contralateral ear under computer control using TDT (Tucker-Davis Technologies) System 2 hardware and custom software. Two electrostatic loudspeakers (TDT-EC1) were driven by two TDT-ED1 modules. The sound system was calibrated using a ¼” condenser microphone (model 4136, Brüel&Kjær) and a dynamic signal analyzer (Photon + , Brüel&Kjær). The maximum sound system output was flat between 0.3–5 kHz (~100 ± 7 dB SPL) and between 5–40 kHz (~90 ± 5 dB SPL). The system’s maximum frequency output was 40 kHz. The second and third harmonic components of the signal were at least 40 dB re maximum output (i.e., lower than the level of the fundamental frequency at the highest output level^17^.

The electrode was advanced using a Sensapex microdrive. Action potentials were recorded with a Bioamp amplifier (Tucker-Davis Technologies; TDT) whose 10X output was further amplified and bandpass-filtered (TDT PC1; fc, 500 Hz and 3 kHz) before passing through a spike discriminator (TDT SD1). Spike times were logged on a computer by feeding the output of the spike discriminator into an event timer (TDT ET1) synchronized to a timing generator (TDT TG6).

Stimulus generation and on-line data visualization were controlled with custom software. Spike times were displayed as dot rasters ordered by the acoustic parameter varied during testing.

### Auditory stimulation

Search stimuli were pure tones or noise bursts. To the extent possible, the approximate frequency tuning of the neuron was determined audiovisually. The minimum threshold and best frequency (BF) of the neuron were then obtained by an automated procedure with 2–5 stimulus repetitions at each frequency and intensity step.

The monaural frequency response area (FRA), i.e., the combination of frequencies and intensities capable of evoking a response, was then obtained automatically using a randomized stimulus presentation paradigm and plotted using Excel, SigmaPlot and Matlab software. The stimuli used to generate FRAs for single units were pure tones with a duration of 75 ms (5 ms rise/fall time). Frequency and intensity of the stimulus were varied randomly (0–100 dB attenuation in 5 or 10 dB steps and in 25 frequency steps from 0.1–40 KHz to cover approximately 2–3 octaves above and below the BF^65^.

Oddball paradigm. We presented trains of 400 stimuli containing two different frequencies (*f*_*1*_ and *f*_*2*_) presented in a pseudo-random order at a specific repetition rate (4 Hz) and at a level of 10–40 dB above threshold. Both frequencies were within the excitatory FRA previously determined for the neuron. One frequency (*f*_*1*_) was presented as standard (i.e., high probability within the sequence, p = 0.9); interspersed randomly with the second frequency (*f*_*2*_) presented as deviant (i.e., low probability within the sequence, p = 0.1). After obtaining one data set, the relative probabilities of the two stimuli were reversed, with *f*_*2*_ as the standard and *f*_*1*_ as the deviant (total number of stimuli for the frequency pair = 400). The frequency contrasts were chosen with variations between 0.14–0.53 octaves, as in previous studies^17^.

### Analysis of neuronal responses (*f*_*1*_ and *f*_*2*_)

The level of SSA for both frequencies at each condition (Common SSA Index, CSI) was calculated as:

$$CSI=\frac{\sum DEV({f}_{i})-\sum STD({f}_{i})}{{\sum }^{}DEV({f}_{i})+\sum STD({f}_{i})};i=1,2$$

where DEV(*f*_*i*_), STD(*f*_*i*_) are spike counts in response to frequency *f*_*i*_ when it was a deviant and standard, respectively. CSI reflects the extent to which the response to the standard stimulus was suppressed. The index ranges between −1 to +1, being positive if the response to the deviant stimulus was greater than the response to the standard stimulus. To test for effects of the drugs on each individual neuron, the 95% confidence intervals (CIs) for the baseline CSI were calculated using a bootstrapping method^39, 68^ (10000 repetitions). The CSI is a special statistic, resulting from a complex combination of the single-trial responses to deviants and standards in a specific way; thus, the sample distribution of CSI for any neuron is highly non-normal, and confidence intervals or standard error of the sample CSI cannot be easily determined using analytical methods. For this reason, we use a bootstrap approach to determine empirical confidence intervals for our sample CSI values, and to assess statistical significance of changes in the neuronal CSI. We have used the bootstrapping method previously^22, 25, 33, 39, 68, 72^, it is very robust and yields accurate results for high resampling size (e.g. 10000 resamplings).

To characterize the time course of adaptation, we plotted the averaged response to the standard frequency from the neurons with significant change after drug application as a function of time. We performed a nonlinear least-square fit to this population mean curve to find the best-fitting double exponential function as follows:$f(t)={A}_{ss}+{A}_{r}\cdot {e}^{-t/\tau (r)}+{A}_{s}\cdot {e}^{-t/\tau (s)}$, where A_ss_, A_r_, and A_s_ are the magnitudes of the steady state and the rapid and slow components, respectively, and τ_(r)_ and τ_(s)_ are the time constants of the rapid and slow components (for details, see ref. 39).

### Spontaneous activity

Drug-induced effects on spontaneous activity were measured by averaging the firing rate over a time window of 150 ms at the end of each sound presentation trial in the control condition and comparing this to that obtained in the same time window during the drug application.

### Drugs

We used two CB1R agonists: anandamide [AEA, N-(2-Hydroxyethyl)-5Z,8Z,11Z,14Z-eicosatetraenamide, Tocris, UK] and O-2545 hydrochloride [(6aR,10aR)-6a,7,10,10a-Tetrahydro-3-(5-{1H-imidazol-1-yl}-1,1-dimethylpentyl)-6,6,9-trimethyl-6H-dibenzo[b,d]pyran-1-ol hydrochloride, Tocris, UK] and one CB1R antagonist: *AM251* [N-(Piperidin-1-yl)-5-(4-iodophenyl)-1-(2,4-dichlorophenyl)-4-methyl-1H-pyrazole-3-carboxamide, Tocris, UK].

The two agonists were applied through different administration pathways due to differences in their water solubility. Microiontophoresis is a technique that can be used only for water soluble drugs. We used anandamide, which is not water soluble, since it is the endogenous agonist of CBR, although it had to be injected into the circulatory system. We administered O-2545, a water soluble compound that is a potent synthetic agonist, by microiontophoresis.

Anandamide and AM251 were administrated through the tail vein^2^ (0.5 mg/kg each). Anandamide was supplied pre-dissolved in anhydrous ethanol and dissolved to a final concentration of 1:19 ethanol/saline. AM251 was dissolved in 1:19 DMSO/saline and sonicated for 30 minutes to make a homogeneous solution. Control experiments were performed using the solution vehicles alone, and no changes were apparent (data not shown).

O-2545 hydrochloride was applied iontophoretically through multi-barreled pipettes attached to the recording electrode so that it was released into the micro-domain of the recorded neuron^25, 39^. The tip of the recording electrode protruded 15–25 µm from the pipette tip. The glass pipette consisted of five barrels in an H configuration (World Precision Instruments, catalog no. 5B120F-4) with the tip broken to a diameter of 30–40 µm^44^. The center barrel was filled with saline for current compensation (165 mM NaCl), whereas the others were filled with 25 mM O-2545. The drug was dissolved in distilled water and its pH adjusted to 3 with HCl. The drug was retained in the pipette with a −20 nA current and was ejected, typically, using 10–20 nA currents (Neurophore BH-2 system, Harvard Apparatus). This drug concentration has been previously demonstrated to be effective in *in vivo* studies in the mammalian visual thalamus^2^. The duration of the drug ejection was usually 5–10 min. After the drug ejection, we repeated the stimulation protocol until we observed recovery.

Recovery of drug application was considered when spike counts returned to levels that did not differ significantly from control values.

### Histological verification of recording sites

At the end of each experiment, electrolytic lesions (5 μA, 5 s) were made with the tungsten recording electrode. Then, animals were sacrificed using a lethal dosis of penthobarbital and decapitated the animal so that brains were immediately fixed using a mixture of 1% paraformaldehyde and 1% glutaraldehyde diluted in 0.4 M PBS (0.5% NaNO_3_ in PBS). After fixation, tissue was cryoprotected in 30% sucrose and sectioned in the coronal or sagittal plane at a thickness of 40 µm on a freezing microtome. Slices were stained with 0.1% cresyl violet to facilitate identification of cytoarchitectural boundaries. The recorded units were assigned to one of the main subdivisions of the IC using as reference the standard sections from a rat brain atlas^66, 69–72^.

### Statistics

Results were analyzed using the *Student t-test* comparing control condition versus drug application and reported as mean ± SE. When data failed to pass the normality test, a non-parametric *Mann-Whitney rank sum test* was performed.

All analyses were done with Sigma Plot software, except bootstrapping which was done using MATLAB.

### Data availability statement

The datasets generated during and/or analyzed during the current study are available from the corresponding author on reasonable request.
